# The Synthesis Followed by Spectral and Calorimetric Evaluation of Stability of Human Milk Fat Substitutes Obtained from Thistle Milk and Lard

**DOI:** 10.1155/2019/5417962

**Published:** 2019-05-02

**Authors:** Joanna Bryś, lnês Filipa Vaz Flores, Agata Górska, Ewa Ostrowska–Ligęza, Andrzej Bryś, Tomasz Niemiec, Piotr Koczoń

**Affiliations:** ^1^Faculty of Food Sciences, Warsaw University of Life Sciences, Nowoursynowska St. 166, 02–787 Warsaw, Poland; ^2^Faculty of Biotechnology, Catholic University of Portugal, Dr. António Bernardino de Almeida St., 4200–072 Porto, Portugal; ^3^Faculty of Production Engineering, Warsaw University of Life Sciences, Nowoursynowska St. 166, 02–787 Warsaw, Poland; ^4^Faculty of Animal Sciences, Warsaw University of Life Sciences, Nowoursynowska St. 166, 02–787 Warsaw, Poland

## Abstract

The central point of current investigations was the first time ever synthesis of modern substitutes of human milk fat followed by versatile evaluation of their oxidative properties. The enzymatic interesterification conducted at 70°C for 2, 4, and 6 hours, respectively, with milk thistle oil and lard blend as starting reactants was catalyzed by 1,3-specific lipase Lipozyme RM IM, obtained from Rhizomucor miehei. Pressure Differential Scanning Calorimetry (PDSC) and Fourier Transform Infrared Spectroscopy (FT-IR) were applied to evaluate quality of products formed. Although PDSC curves showed lower oxidative stability of newly synthesized fats as compared to both starting materials separately, they can be considered adequate substitutes of human fat milk fat, as distribution of fatty acids in triacylglycerol molecules of substitutes obtained is much alike human milk fat itself, as resulted from analysis of GC data collected. Obvious changes in chemical structure of fats occurring during interesterification resulted in specific alterations in IR spectra of processed materials. Spectral data accompanied by PLS technique were successfully used for accurate determination of oxidative stability of new fats through indirect procedure, i.e., IR-PDSC-reference analysis of induction time. Additionally IR data exclusively, i.e., without any reference data, occurred powerfully in discrimination of human fat milk substitutes obtained.

## 1. Introduction

The structure of main constituents of food (the so-called food matrix) plays a vital role in the process of food interaction with chemicals present in human digestive track. This results in different uptake/release of nutrients contained in given food. The major form of lipids in foods is TAG that consists of three fatty acids attached with ester bonds to carbon chain of glycerol backbone. Chemical structures of fatty acids, i.e., carbon chain length and degree of saturation, together with their location position in glycerol (sn-1, sn-2, or sn-3), determine chemical, physical, and biological properties of TAG. This results in various digestibility followed by different impact on human health when fats are consumed [1].

Human milk is the only food for infants. Energy of chemical bonds of lipids contained in human milk is the main source of energy for newborn. The chemicals provided with fats are also crucial structural component used in formation of membranes of new cells [2]. The fatty acid composition of HMF is very unique. It is of high content of the saturated palmitic acid and contains specific polyunsaturated fatty acids that are absent in other fats, i.e., fats contained in milks of different origin [2–5]. The TAG structure of human milk is unique as well. Approximately 65% of palmitic acid is located at the sn-2 position. Furthermore, 18:0, 18:1, and 18:2 fatty acids are mainly located at sn-1 and sn-3 positions, respectively [5, 6]. The gastric lipase in the stomach and pancreatic or bile salt-stimulated lipases catalyze formation of body-required “breaks” from fat absorbed from small intestine; thus structure of fat absorbed much influences the structure of final product [2].

The saturated fatty acids are located almost exclusively at the 1 and 3 positions in vegetable oils TAG. Animal originated fats, e.g., tallow or lard, are almost exclusively equipped with saturated fatty acids at the sn-2 position [1]. The lard has been recommended as an alternative to HMF due to much alike HMF structure in terms of fatty acids composition and their distribution in TAG [7]. Compared to HMF, the content of palmitic and oleic acids in lard is at same level while the content of essential fatty acids is significantly lower [4].

Silybum marianum L. Gaertn. (synonym: Carduus marianus L.), commonly known as milk thistle, is a member of the Asteraceae family. It is a native plant from the Mediterranean area that is an important industrial agricultural crop [8, 9]. Milk thistle has been studied asa hepato-, neuro-, nephron-, and cardioprotective and anti-inflammatory, antivirus agent mainly due to its strong antioxidant properties. This plant has absorbed much attention due to its antihyperglycemic and hypoglycemic properties in diabetes mellitus [10]. Seeds of milk thistle are known to be used for more than 2000 years to treat liver diseases [9]. Poland is an important European producer of milk thistle seeds and medicines derived from it. Cultivated area covers about 2000 ha [9, 11]. The compounds of medicinal value are contained in the seeds of this plant. They contain silymarin and 25% (w/w) of oil [9, 12]. Silymarin is well known as antioxidant and have been used for the treatment of cancer, liver, kidney, cardiac, brain, cirrhosis, and poisoning of alcohol, drugs, or toxins [9, 13–15]. The milk thistle seeds contain unsaturated fatty acids, e.g., oleic acid or linoleic acid [15].

Interesterification is well known procedure for modification of physicochemical properties of oils and fats. Within this process fatty acids are exchanged within and among TAG until a thermodynamic equilibrium is reached [7, 16, 17]. The interesterification can be conducted either chemically or enzymatically. The alkaline catalysts, such as sodium alkoxide and sodium hydroxide, are usually applied as homogeneous catalysts in vegetable oils interesterification [18, 19]. As compared to the chemical catalysts, the lipasea biocatalyst has many merits including milder reaction conditions, selectivity, and ease of product recovery [19, 20]. The use of 1,3-selective lipases allows to maintain the fatty acids in the sn-2 position of the acylglycerols, that is nutritionally desirable and impossible by chemical catalysis [21, 22]. The enzymatic synthesis of HMF alike fats as well as interesterification using specific sn-1,3 lipases are considerable strategies [2, 7, 23, 24].

Although an interest from both market and science about HMF continuously increases, no fat of identical or fully acceptable HMF alike properties has been obtained so far. Therefore the aim of current study was to synthesize and evaluate the selected properties, including OS, of HMFS. Synthesis method was enzymatic interesterification of a blend of lard and milk thistle. It was assumed that a combination of lard and milk thistle in interesterification process opens possibility of obtaining HMF alike fat in terms of the fatty acid composition and their distribution in TAG [2, 25, 26]. To thoroughly evaluate properties of newly synthesized fats, thus achieve stated aim, analytical data obtained by PDSC and FT-IR techniques were processed. The analytical information obtained from thermal analysis is useful in controlling quality changes in food during processing and storage [27–29]. DSC is a physical method to determine fat quality parameters, especially OS of oils and fats [7, 30–33]. IR spectroscopy serves as an established method to determine types and number of chemical bonds present in studied sample and therefore can be used to follow chemical changes occurring in processed fats [29, 31].

## 2. Materials and Methods

The mixture of initial materials, i.e., lard and milk thistle oil (8:2 w/w) obtained from “Zakład Mięsny Wierzejki” (Poland) and “PPHU Maszyny i Przetwórstwo Nasion Oleistych Ol'Vita” (Poland), respectively, were interesterified. Lipozyme RM IM preparation that contained immobilized lipase from* Rhizomucor miehei *was used as biocatalyst. Reaction was conducted in 70°C for 2, 4, and 6 hours, respectively, in thermostatic shaker. The temperature incubation of substrates lasted for 10 minutes and was followed by addition of biocatalyst (8% mass to mass, heterogenous catalysis) that was considered an onset point of interesterification reaction. Then, after desired time reaction was stopped by separation of biocatalyst, with use of Buchner funnel under reduced pressure.

Samples obtained were then analyzed with GC due to fatty acids composition and their distribution between three positions in glycerol backbone. First TAG underwent saponification and esterification with methanol to obtain methyl esters. As pork pancreas lipase has ability to select hydrolysis of esters in sn-1,3 position TAG were hydrolyzed with use of this specific enzyme. Products of this hydrolysis were extracted with diethyl ether. Then preparative thin-layer chromatography was used to separate dissolved in ether products of enzymatic diacylation of TAG. Isolated this way sn-2 monoacylglycerols were removed from plates together with gel and eluted with diethyl ether. The fatty acids contained in monoacylglycerols were determined with GC technique. Fatty acids composition and their positional distribution in sn-2 and sn-1,3 positions of TAG were determined as described further [5, 7, 29].

Two substantial fat features, i.e., peroxide value and acidic value, were determined by classical titration according to the ISO standards 3960:2007 and 660:2009, respectively, for both raw and interesterified materials. Data obtained were statistically processed with application of one-factor variance with Tukey's test at significance level *α*= 0.05.

### 2.1. DSC Measurements

OS was evaluated by data obtained with a differential scanning calorimeter (DSC Q20 TA) coupled with a high-pressure cell. Given sample was precisely weighted in dedicated aluminium pan. Then the pan with fat was moved to measuring chamber. Chamber was filled with oxygen and initial pressure was set to 1 400 kPa. The isothermal temperature for each sample was 120°C. TA Universal Analysis 2000 software was applied to analyze obtained diagrams.

### 2.2. FT-IR Measurements

Infrared spectra were recorded with use of Perkin Elmer System 2000 spectrometer in middle range, i.e., 4000–400 cm^–1^. The resolution was set at 2 cm^–1^. Drop of fat of 0.05 cm^3^ volume was placed between ZnSe plates to form film. Instrument was operated by Pegrams software running on Windows 95.

## 3. Results and Discussion

DSC experiments are performed in dynamic conditions that means the linear increase in temperature is applied or at isothermal conditions that means constant temperature is applied. The pressure of oxidation medium, i.e., oxygen or air, is kept either at ambience pressure or at increased pressure (PDSC) [35]. In current studies to determine OS of material investigated induction time was measured.

**Figure 1:**
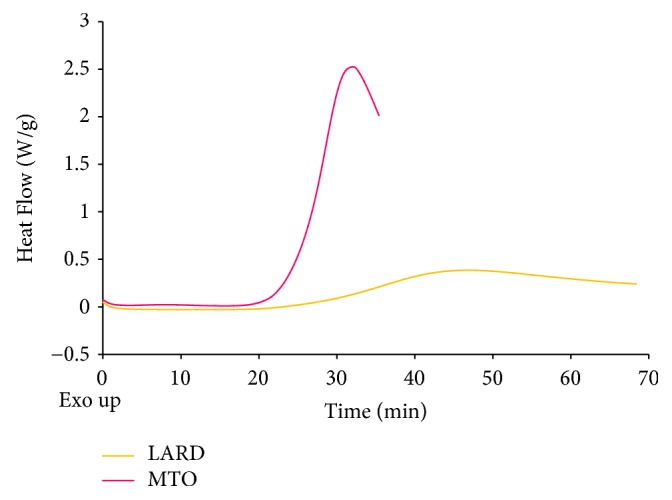
PDSC curves of MTO and L.

**Figure 2:**
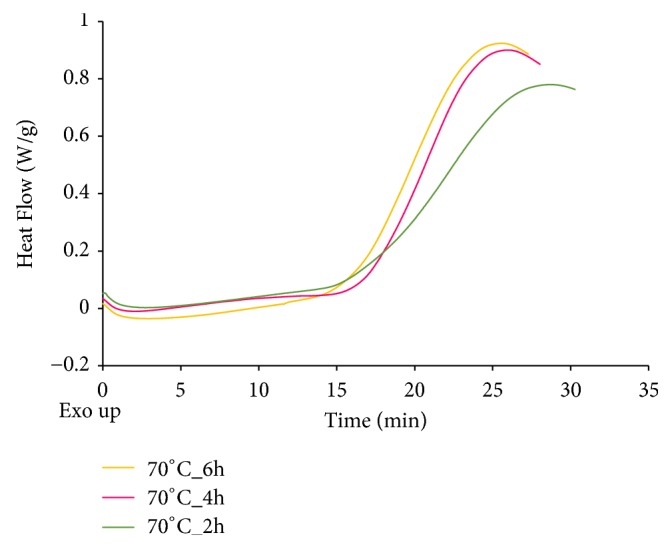
PDSC curves of HMFS obtained after 2-, 4-, and 6-hour interesterification.

**Figure 3:**
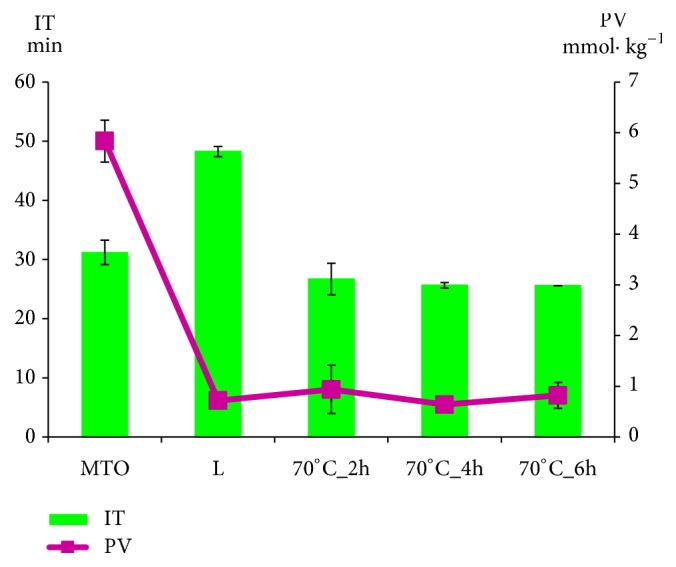
Oxidation induction time (IT, green bars) and peroxide value (PV, violet points) of L, MTO, and HMFS obtained by esterification conducted for 2, 4, and 6 hours, respectively.

Figures 1 and 2 present curves of PDSC and Figure 3 presents the values of induction time registered for L, MTO, and HMFS obtained in different time of interesterification with PDSC technique. Samples with smaller induction time are less stable compared to those with greater induction time, considering results obtained at the same temperature [36]. For HMFS samples induction time measured isothermally at 120°C ranged from 25.6 min to 26.7 min. It was smaller comparing to the initial fats for which results were as follows: MTO-31.2 min; L-48.2 min. Most literature records report a decrease in OS of interesterified fats compared to the initial mixture [29, 37, 38]. OS is considered primary factor determining the overall quality of foodstuff of high content of unsaturated fats and oils as well as fats and oils themselves. Polyunsaturated fatty acids are chemically highly reactive molecules due to high content of reactive double bonds and hence susceptibility for oxidation, that results in formation of free radicals, hydroperoxides, and polymers. This in turn leads to decrease in quality, both technological and nutritional [38]. Induction time measured for lard samples was longer than that measured for milk thistle oil. The long induction time, hence greater OS of lard, is the result of high content of saturated fatty acids. PV of lard was also smaller than that of milk thistle oil. PV determines the current amount of oxygen chemically bounded to an oil or fat, i.e., involved in peroxides. Particularly hydroperoxides, the primary products of oxidation, are significant. Higher value of PV indicates a higher production of primary oxidation products which leads to quicker progression of oxidation. In Figure 3 the PV of L, MTO, and HMFS are presented. The PV values were significantly lower for HMFS as compared to raw materials. The PV values of products of enzymatic esterification were lower than 1 mmol O_2_ · kg^–1^ fat, which means that they are resistant to oxidation.

**Figure 4:**
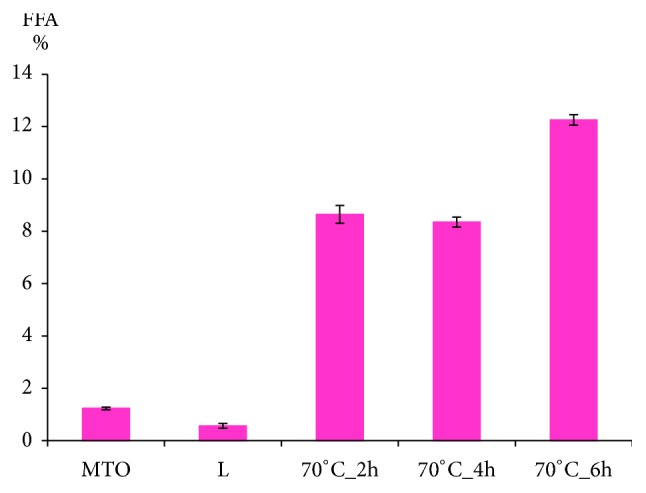
Free fatty acids (FFA) content in L, MTO (raw materials), and HMFS obtained after 2-, 4-, and 6-hour interesterification, respectively.

The ordinary function of lipases is to catalyze the process of hydrolysis of fats to form FFA, partial acylglycerols and glycerol. In the case of reversible reaction the enzyme catalyzes both forward and reverse reactions, i.e., the back formation of acylglycerols from glycerol and FFA. Depending on level of water equilibrium shifts to the reactants that means hydrolysis predominates or to products thus esterification predominates [39]. Hence, FFA can be present in the reacting mixture. Hamam and Shahidi [40] suggested that FFA may induce oxidation due to catalytic effect of the carboxylic groups on formation of free radicals. The FFA content in lard, milk thistle oil, and HMFS is shown in Figure 4. The content of FFA in lard was the lowest, i.e., <1%. The content of FFA in milk thistle oil was higher than in lard but numerically lower than 2%. On the other hand, the content of FFA in HMFS was greater compared to both lard and milk thistle oil. These results are in agreement with Bryś et al. [7], who proved that enzymatic interesterification leads to increase in FFA content. The FFA content depends significantly on the duration of the process. In the case of HMFS the shorter reaction time the lower FFA content. The final mixture obtained after lard and milk thistle oil interesterification contained 8.3–12.3% of FFA. Before using HMFS as functional food additive, the FFA should be removed.

**Figure 5:**
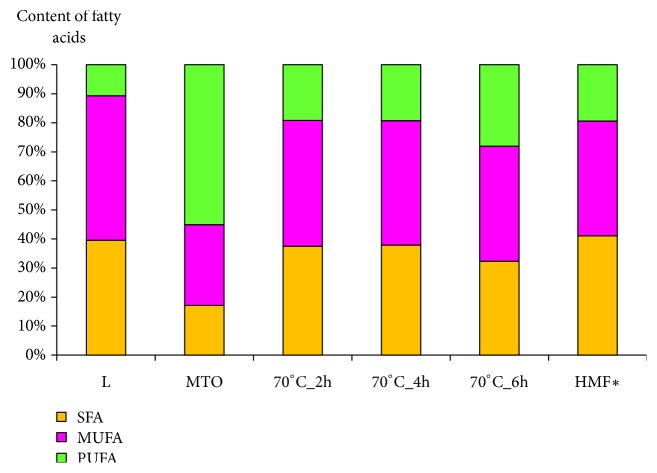
Content of fatty acids (SFA saturated fatty acids, MUFA monounsaturated fatty acids, PUFA polyunsaturated fatty acids) in L, MTO, mixtures after interesterification and in human milk fat. **∗** Human milk fat. Values are derived from Lopez–Lopez et al. [34].

The chemical composition, i.e., presence of required fatty acids of initial blend, is essential for the formation of proper HMFS by the interesterification [7, 23]. As shown in Figure 5 these mixtures of lard and milk thistle oil used for esterification were appropriate to produce HMFS due to ratio of PUFA to MUFA and SFA similar to adequate ratio in HMF. The SFA content in HMFS ranged from 32.3 to 37.9%, the MUFA from 39.6% to 43.3%, and PUFA from 19.1% to 28.0%. Some of unsaturated fatty acids present in milk thistle oil were transferred to backbone of lard substituting some saturated acids. The content of both oleic and palmitic acid in obtained HMFS is similar to the adequate ratio in HMF. TAG of interesterified fats contained from 17.7% to 22.2% of palmitic acid and from 37.1% to 40.3% of oleic acid. HMF contains 40.6% of SFA including about 22% palmitic acid and 39.1% of MUFA including about 34% oleic acid [34]. As a result of interesterification the essential fatty acids from milk thistle oil were built-in structure of TAG of lard. The obtained HMFS contain from 19.1% to 28.0% of PUFA including omega-3 and omega-6 fatty acids. Therefore the interesterification of a blend of lard and milk thistle oil forms new fats that are of similar to HMF oleic and palmitic acids content, as well as containing nutritionally important essential fatty acids from the omega-6 and omega-3 groups.

**Figure 6:**
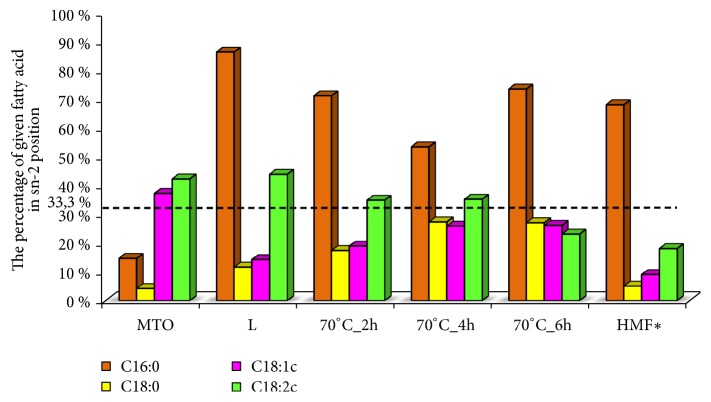
The percentage of a given fatty acid in sn-2 position of TAG of lard, milk thistle oil, and interesterified fats. *∗*Human milk fat. Values are derived from Lien et al. [3].

HMF is believed to be a model for the fat components in infant formulas [2, 41]. Human milk contains approximately 70% of palmitic acid in the sn-2 position, whereas most vegetable oils contain this specific fatty acid primarily in the sn-1 and sn-3 positions [3, 6]. Pancreatic lipase selectively hydrolyzes the fatty acids at the sn-1 and sn-3 positions, yielding FFA and MAG. The 2-MAG are absorbed in digestive track more efficiently than free palmitic acid, which form insoluble salts with calcium and magnesium cations [2, 42]. In Figure 6 the percentage of most abundant fatty acid in sn-2 position of TAG of lard, milk thistle oil, and HMFS are presented. HMFS, alike HMF, contains from 53.4% to 73.4% of palmitic acid in the sn-2 position of TAG, whereas percentage of unsaturated fatty acids in internal position of TAG in oil is from 18.9% to 26.1% and from 23.0% to 34.8% for oleic and linoleic acid, respectively. In regard to the percentage of the fatty acids esterified at sn-2 position of TAG in HMFS, one can observe that the structure of TAG of HMFS is very alike in the structure of HMF with respect to fatty acids present in sn-2 position.

**Figure 7:**
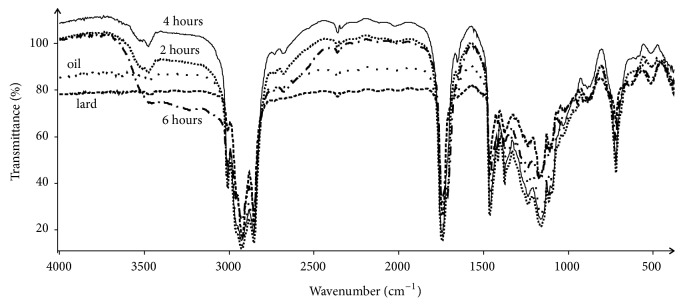
Raw averaged spectra of by lard, milk thistle oil, and 2-, 4-, and 6-hour temperature (70°C) treated mixtures (lard: oil, 8:2). Different line styles are for different samples. Transmittance is plotted against wavenumber.

Raw spectra of studied samples are presented on Figure 7. They are quite similar to each other with a very strong bands (high transmittance) around 3000 cm^–1^ and 1750 cm^–1^, respectively. Water-free samples are appropriate materials for IR analysis as water absorbance generated by IR very active O–H vibration does not disturb or influence spectral effect of oscillating atoms contained in specific groups, e.g., functional groups of studied compounds. Presented spectra are similar to each other as all are of similar origin and contain similar groups of vibrating atoms that results in similar spectral shape. Direct analysis of wavenumbers difference or intensity difference or bands shift is difficult. Therefore in current approach visual inspection was followed by statistical techniques application.

**Figure 8:**
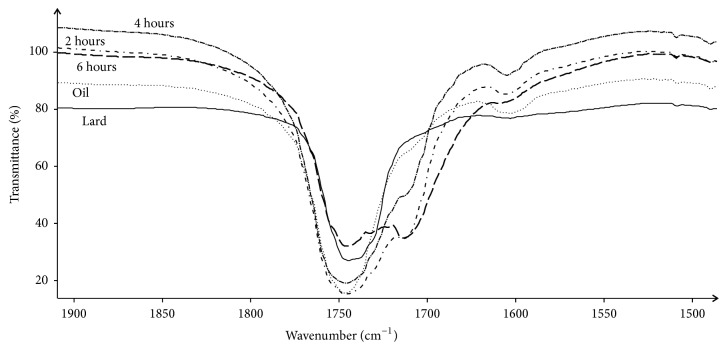
Transmittance plotted against wavenumber for lard, oil, and 2-, 4-, and 6-hour temperature (70°C) treated mixtures (lard: oil, 8:2). Extension in 1900–1550 cm^–1^ spectral region. Different line styles denote different samples.

Spectra of samples of lard, oil extracted from thistle, and temperature treated 8:2 mixtures of both were analyzed due to their similarities and differences. Spectral data in some regions differed significantly. In following regions, 3200–2800 cm^–1^ containing bands originated from C–H oscillations, 1800–1600 cm^–1^ in which bands are generated mainly by C=O vibrations affected by configuration of groups of atoms attached to carbonyl carbon, and 1480–1450 cm^–1^, differences can be observed even directly with visual inspection after computer extension of these regions.  Figure 8 presents example spectral data of extension in 1900–1550 cm^–1^ region. This is very characteristic region for fat-like materials as contain bands originated from C=O vibrations contained in carboxylic groups, ester groups, carbonyl groups, or even amide groups. All spectra have very strong and sharp band at 1746 cm^–1^. The less intense but still strong band located at 1712 cm^–1^ is present in the case of spectra of interesterified mixtures only. Lard and oil do not have this band which evidences for creation a new chemical structure during interesterification process. 6-hour processed sample has strongest band at this wavenumber.

Spectral differences occur in the range 14761450 cm^–1^. In spectra of 2-hour temperature treated mixtures the middle intensity band at 1466 cm^–1^ accompanied by weak band at 1459 cm^–1^ is present. In the spectrum of 4-hour treated mixtures band at 1466 cm^–1^ is still present, while new band at 1457 cm^–1^ occurs that is considered shifted band observed at 1459 cm^–1^ in spectra of 2-hour temperature treated samples. The shift toward lower frequency evidences that bond generating this band is slightly weaker than original one. In the spectrum of 6-hour temperature treated sample band at 1466 cm^–1^ is present at the same wavenumber, while band at 1457 cm^–1^ becomes more distinct, i.e., is of greater intensity.

Next interesting change observed in spectra of time-differently treated mixtures is band at 1099 cm^–1^. It is clearly present for 2-hour treated sample while it weakens for 4-hour sample and disappears for 6-hour sample. Similar alteration is observed for band at 1030 cm^–1^ that is present in 2- and 4-hour samples while absent in 6-hour sample. On the other hand bands at 1047 cm^–1^, 943 cm^–1^ and 634 cm^–1^ are present in 6-hour sample while absent in 2- and 4-hour samples. All the mentioned above assignments are presented in Table 1.

**Figure 9:**
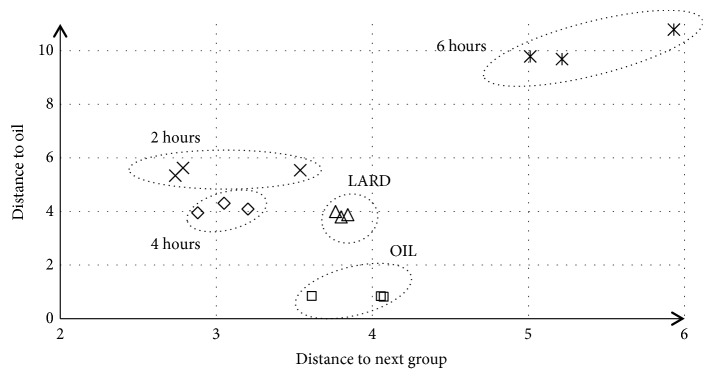
The homologous groups formed by lard, oil, and 2-, 4-, and 6-hour temperature (70°C) treated mixtures (lard: oil, 8:2) obtained by PCA analysis of IR spectra of studied samples.

The differences in spectra of lard, oil, and temperature mixtures are obviously due to difference in their chemical compositions. Discriminant analysis of spectra of five groups resulted in separation of five homologous groups presented graphically in Figure 9. Groups are located in Cartesian system with “distance to next group” on x-axis and “distance to oil” on y-axis. The selection of data on x- and y-axis aimed at clear graphical presentation for distinct diversity of homologous groups. They are statistically far away from each other; however 6-hour temperature treated mixtures are most distinct that is due to bigger chemical changes that occurred during longer time of esterification process.

**Figure 10:**
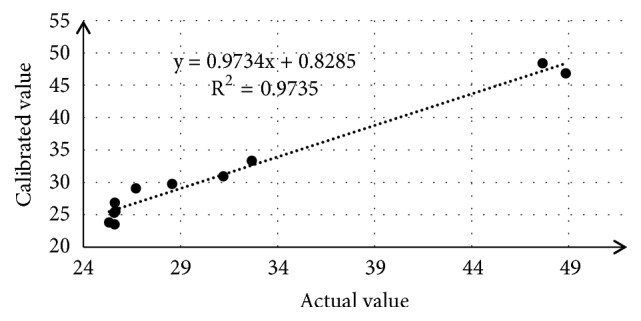
The plot shows the relationship between actual values and calculated values of induction time obtained with use reference model related of spectral dates.

The application of spectral data is versatile. Within current investigation they were referred to results obtained by analysis of different methods data, e.g., DSC. As induction time (IT) is most important parameter of studied samples due to their resistance to oxidation, i.e., OS, its average value determined with DSC technique has been statistically referred to spectral data from selected spectral regions. Reference model has been calculated with use of calibration and validation data sets.  Figure 10 presents dependence between actual and calculated with the model values of IT. The application of different regions as well as combining of several regions did not produce significantly better results in terms of correlation coefficients of validation and prediction of RMSEC and RMSEP. In current model correlation coefficient for linear calibration was 0.9735 with RMESC = 1.36 and RMSEP = 5.06. The linear equation between predicted by model against actual values is y = 0.9734 x + 0.8285. Spectral region used for model calibration was 3115–2737 cm^–1^ of which contribution was 98.7807% while whole spectrum contribution was 98.9806. Model proposed, considered the best, is calibrated with use of 4 factors.

## 4. Conclusions

The mixtures of L and MTO during interesterification process formed HMFS with much similar to HMF fatty acids composition. The distribution of fatty acids in synthesized HMFS is also similar to that in HMF that was primary aim of current studies. However, interesterification process decreased the induction time of HMFS as compared to starting materials, which was suggested to be due to relatively high level of FFA in final product. It was further suggested that using HMSF as functional foods nutritional additives should be preceded by removal of at least part of FFA. PDSC and FT-IR occurred to be fast and reliable method that can be used to assess the oxidative sensitivity features of interesterified mixtures of lard and milk thistle oil. The induction times that can be used as primary parameter of the resistance of fats to their oxidative decomposition were measured with PDSC and correlated with spectral data by PLS technique. Chemical changes occurring during interesterification resulted in many alterations in IR spectra due to breaking and formation of chemical bonds. The information on these alterations can be used for discriminant and reference analysis. PCA analysis constructs statistical model that can be used to distinguish and assign unknown samples and to monitor conditions of process the samples were treated with, e.g., duration of interesterification. The explanation of all abbreviations is given in Table 2.

## Figures and Tables

**Table 1:** The wave numbers of selected bands occurring in spectra of studied compounds.

Spectral region	Samples studied									
	*oil*		*lard*		*2 hours mixture*		*4 hours mixture*		*6 hours mixture*	
	VN*∗*/cm^–1^	Intensity	VN/cm^–1^	Intensity	VN/cm^–1^	Intensity	VN/cm^–1^	Intensity	VN/cm^–1^	Intensity
3200–2800	3010	vs	3010	vs	3010	vs	3010	vs	3010	vs
	2058	vs	2058	vs	2058	vs	2058	vs	2058	vs
	2928	vs	2928	vs	2928	vs	2928	vs	2928	vs
	2855	vs	2855	vs	2855	vs	2855	vs	2855	vs

1800 –1600	1746	vs	1746	vs	1746	vs	1746	vs	1746	vs
					1712	s	1712	s	1712	s

1480 – 1450					1466	m	1466	m	1466	m
					1459	vw	1457	w	1457	m

< 1100					1099	m	1099	w	–	
					1030	m	1030	w	–	
					–		–		1047	w
					–		–		943	w
					–		–		634	w

*∗* VN: wave number, vs: very strong, s: strong, m: medium, w: weak, and vw: very week.

**Table 2:** Abbreviations and symbols.

Abbreviations	Meaning
70°C_2h	The mixture of L and MTO in proportions 8:2, interesterified for 2 h at 70°C

70°C_4h	The mixture of L and MTO in proportions 8:2, interesterified for 4 h at 70°C

70°C_6h	The mixture of L and MTO in proportions 8:2, interesterified for 6 h at 70°C

DSC	Differential Scanning Calorimetry

FFA	Free Fatty Acids

FT–IR	Fourier Transform Infrared Spectroscopy

GC	Gas Chromatography

HMF	Human Milk Fat

HMFS	Human Milk Fat Substitutes

L	Lard

MAG	Monoacylglycerols

MTO	Milk Thistle Oil

OS	Oxidative Stability

PDSC	Pressure Differential Scanning Calorimetry

PV	Peroxide Value

TAG	Triacylglycerols

## Data Availability

The data used to support the findings of this study are included within the article or available from the corresponding author upon request.
